# FDG avid pulmonary amyloid nodule in a patient with metastatic renal cell cancer on 18F-FDG PET/CT

**DOI:** 10.1016/j.radcr.2021.11.029

**Published:** 2021-12-09

**Authors:** Marco Enoch Lee, Veronica Chi Ken Wong, Chuong Bui, Robert Mansberg

**Affiliations:** a Department of Nuclear Medicine and PET, Nepean Hospital, Derby St, Kingswood, NSW, 2747; b Faculty of Medicine and Health, University of Sydney, Camperdown, NSW, Australia, 2006; c Faculty of Medicine and Health, University of New South Wales, Sydney, NSW, Australia, 2052

**Keywords:** Multiple primary neoplasms, Pulmonary amyloidosis, Bowel cancer, Renal cell cancer, 18F-FDG PET/CT

## Abstract

A 60-year-old man with a background of resected clear cell renal cancer and resected colorectal adenocarcinoma presented with a pulmonary mass lesion in the left upper lobe which was avid on 18-F FDG PET/CT. Needle biopsy confirmed metastatic renal cell cancer, which was surgically excised with wedge resection. Follow-up imaging 6 months later demonstrated a second slowly enlarging subcentimeter nodule in the contralateral lung with increasing FDG avidity, suspicious of further small volume oligometastatic disease. Following surgical resection of the second pulmonary lesion, histopathological examination demonstrated nodular pulmonary amyloidosis and no evidence of malignancy.

## Introduction

Amyloidosis encapsulates a group of diseases which involve the deposition of misfolded, insoluble, polymeric protein fibrils which ultimately result in damage to the surrounding tissue or organ. Solitary pulmonary amyloidomas are great mimickers of malignancy and should be considered in a patient with a pulmonary nodule even in the setting of metastatic disease. We present a case of a solitary pulmonary amyloidoma mimicking metastases in a patient with previous proven pulmonary metastatic disease from renal cell cancer.

## Case presentation

**Fig. 1:**
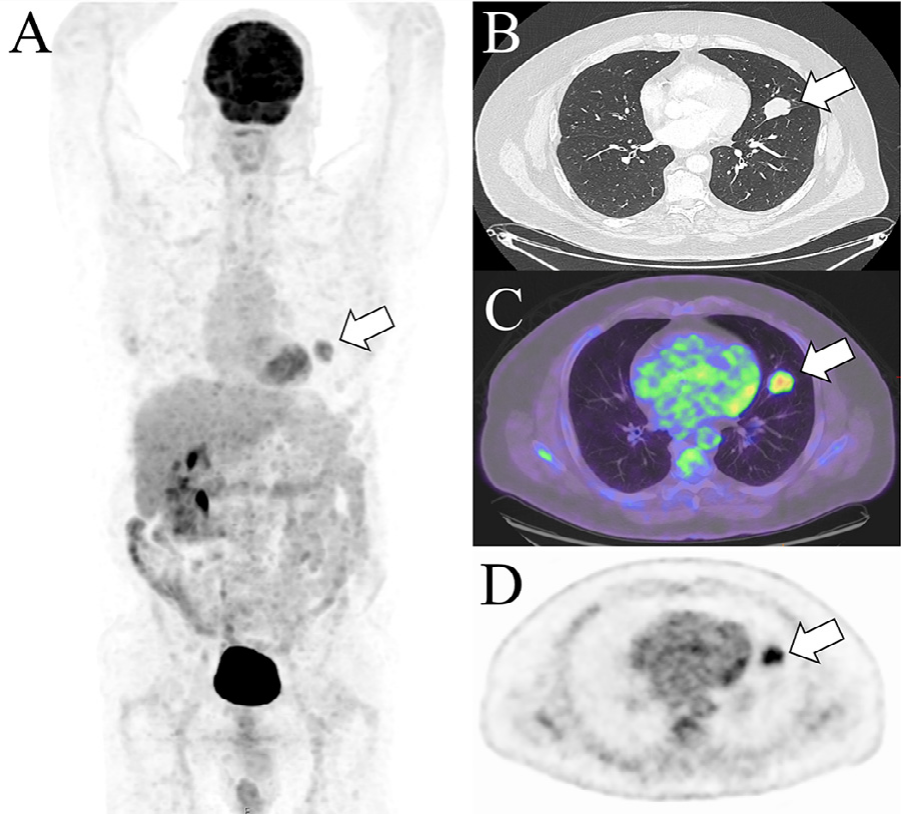
Transaxial CT (B, post-contrast) revealing a 2.9 cm slightly lobulated lesion in the inferior lingular segment of the left upper lobe overlying the left oblique fissure (white arrow). On 18-F FDG PET/CT, maximum intensity projection (A), transaxial fused PET/CT (C) and transaxial PET (D) images demonstrated moderate to marked FDG avidity (SUVmax 6.4) in this lesion (white arrows).

A 60-year-old man with history of left nephrectomy for clear cell renal cancer (stage I) and resected colorectal adenocarcinoma (stage II) presented with a pulmonary mass lesion in the left upper lobe on surveillance imaging. Transaxial CT (Fig. 1B) revealed a 2.9 cm slightly lobulated lesion in the inferior lingular segment of the left upper lobe overlying the left oblique fissure. 18-F FDG PET/CT images (Fig. 1A, C, D) demonstrated moderate to marked FDG avidity (SUVmax 6.4) in this lesion.

Histopathology of CT-guided core needle biopsy showed tumour cells with relatively uniform nuclear and clear cell cytoplasm with positive staining for PAX8 and renal cell antigen, consistent with metastatic renal cell carcinoma. The patient underwent wedge resection of the left upper lobe metastasis with clear excisional margins.

**Fig. 2:**
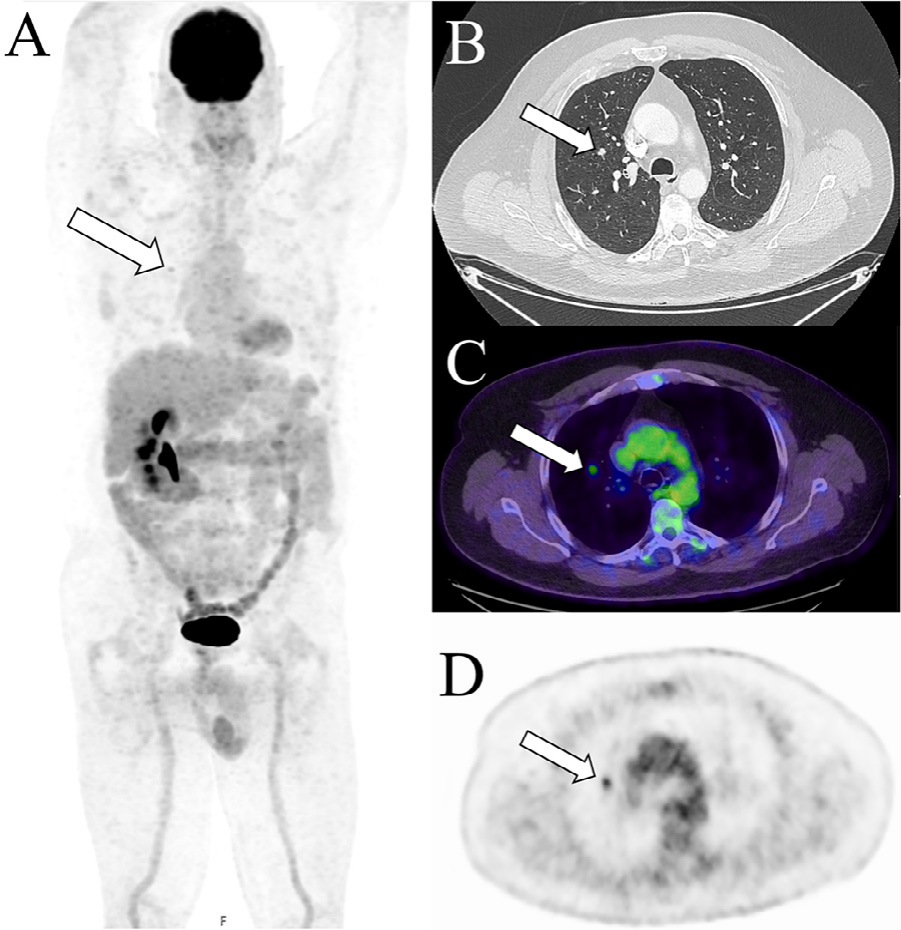
Transaxial CT (B, post-contrast) demonstrating a slowly enlarging subcentimeter 9mm pulmonary nodule in the right upper lobe (white arrow). Maximum intensity projection (A), transaxial fused PET/CT (C) and transaxial PET (D) images demonstrated moderate FDG avidity (SUVmax 4.6 on current PET, SUVmax 2.4 on previous PET (not shown)) in this lesion (white arrows).

6 months later, follow-up imaging with 18-F FDG PET/CT and diagnostic CT was performed. Transaxial CT (Fig. 2B) demonstrated a slowly enlarging subcentimeter 9mm pulmonary nodule in the right upper lobe. Maximum intensity projection, transaxial fused PET/CT and transaxial PET (Fig. 2A, C, D) images demonstrated moderate FDG avidity (SUVmax 4.6 on current PET, SUVmax 2.4 on previous PET (not shown)) in this lesion. No other suspicious FDG avid focus was detected. Given the proven pulmonary metastatic disease from renal cell cancer, further small volume oligometastatic disease was suspected. Reliable needle biopsy of the lesion was considered difficult due its small size and deep location. The patient subsequently underwent segmental resection of this sub centimeter pulmonary nodule.

**Fig. 3:**
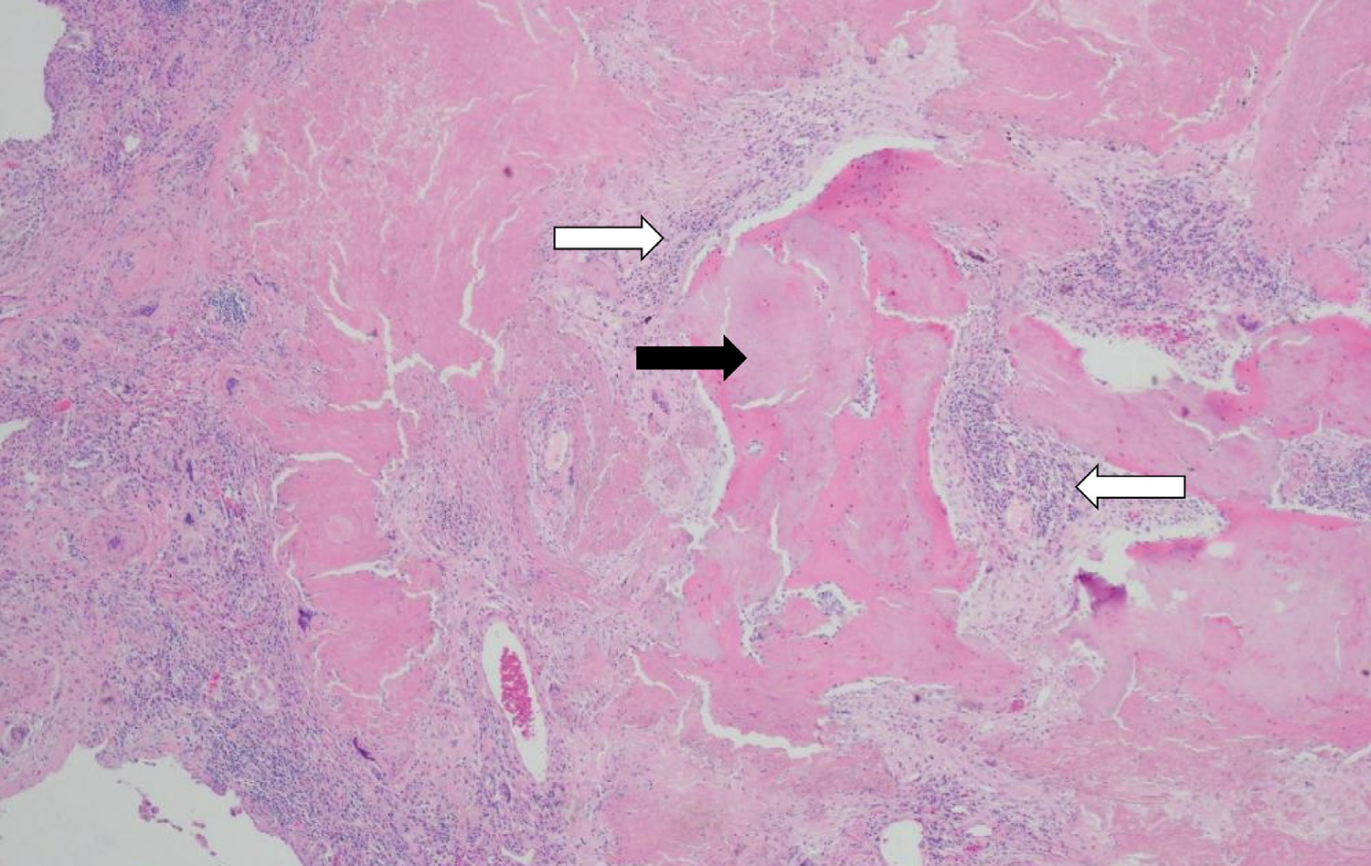
Histopathological sections showed amorphous hyaline acellular amyloid-like material (black arrow) associated with chronic inflammatory cells eliciting a foreign body multinucleated histiocytic cell response (white arrows). The chronic inflammatory cells are composed of mature plasma cells with smaller numbers of lymphocytes. Congo red stain shows focal characteristic birefringence and crystal violet stain is strongly and uniformly positive, confirming amyloid.

Histopathology of the second pulmonary nodule demonstrated no evidence of malignancy. Instead, there was amorphous hyaline acellular amyloid-like material associated with a rim of multinucleated inflammatory cells (Fig. 3). The chronic inflammatory cells comprised mainly of mature plasma cells with smaller numbers of lymphocytes. Crystal violet stain was positive, and the Congo red stain demonstrated focal birefringence, confirming nodular pulmonary amyloidosis. The patient continues to be under observation for further tumour recurrence.

## Discussion

Amyloidosis encapsulates a group of diseases which involve the deposition of misfolded, rigid, polymeric protein fibrils which ultimately result in damage to the surrounding tissue or organ [1]. This can be a localised or systemic process involving any organ system within the human body, including the lungs [2]. Primary pulmonary amyloidosis is uncommon and solitary thoracic amyloidomas are rare [3,4].

Although misfolded amyloid proteins are not metabolically active, plasma cells producing amyloid proteins and the resultant inflammatory response are metabolically active. This results in variable FDG avidity within the pulmonary nodule, acting as a mimicker of primary pulmonary malignancy [5], [6], [7], [8]. In the setting of previous malignancy, amyloidosis can imitate metastatic disease.

Delineation between malignancy and amyloidosis with evaluation of morphological appearance and metabolic activity on imaging is often difficult. Dual phase FDG PET/CT has been described as a technique to delineate between malignancy and amyloidosis [9], although this is not current routine practice. Biopsy with histopathological diagnosis remains the most reliable tool to distinguish a focal amyloidoma from metastasis. Amyloidomas microscopically show acellular hyaline material which demonstrate characteristic birefringence on Congo red stain [10].

## Conclusion

This case study demonstrates that localised nodular amyloidosis is uncommon but may act as a great mimicker of malignancy. Amyloidosis should be a consideration in patients with enlarging FDG avid pulmonary nodules even in the setting of previous pulmonary metastatic disease.
